# An atlas of colonization factors in the mouse gut microbiome reveals phylogenetically structured repertoires and infection-stage-associated variation during *Toxoplasma gondii* infection in mice

**DOI:** 10.1128/spectrum.01940-26

**Published:** 2026-09-22

**Authors:** Yao Zhang, Hany M. Elsheikha, Wen-Bin Zheng, Fu-Long Nan, Rui Liu, Xiao-Xuan Zhang, Li-Hua Yang, Ying Li, He Ma

**Affiliations:** 1 College of Life Sciences, Changchun Sci-Tech University, Shuangyang, Jilin Province, People's Republic of China; 2 College of Food and Pharmaceutical Engineering, Wuzhou University, Wuzhou, Guangxi Zhuang Autonomous Region, People's Republic of China; 3 College of Veterinary Medicine, Qingdao Agricultural University, Qingdao, Shandong Province, People's Republic of China; 4 Faculty of Medicine and Health Sciences, School of Veterinary Medicine and Science, University of Nottingham, Sutton Bonington Campus, Loughborough, United Kingdom; 5 Laboratory of Parasitic Diseases, College of Veterinary Medicine, Shanxi Agricultural University, Taigu, Shanxi Province, People's Republic of China; Sichuan University, Chengdu, China

**Keywords:** colonization factors, mouse gut microbiome, phylogenetic structure, *Toxoplasma gondii*, microbial functional profiling

## Abstract

Colonization factors (CFs) comprise CF-associated gene families related to microbial establishment, persistence, host interaction, and gut ecological fitness, but their organization and variation in the mouse gut microbiome remain poorly defined. We integrated the Mouse Gut Genome Catalogue and the Mouse Gut Protein Catalogue, clustered at 90% amino acid identity, to define CF repertoires. A total of 79 conserved CF families, comprising over 2.4 million homologs, were identified across 112,951 genomes from 2,824 microbial species. Species-level CF-associated gene profiles exhibited a clear phylogeny-associated pattern and separated microbial species into two groups with distinct genome-scale functional profiles. Across the cecum and small intestine, CF profiles differed among control, acute, and chronic infection groups in dominant CF abundance, diversity, richness, and overall composition. CF profiles were significantly concordant with taxonomic profiles and associated with microbial community variation. Differential abundance analyses identified tissue- and stage-associated CF features. Exploratory machine-learning analyses showed high classification performance across all feature sets in the cecum, with the genus-level model yielding the highest median performance, whereas species- and genus-level taxonomic profiles provided stronger discrimination than CF profiles in the small intestine. This study provides a reference-based atlas of CF-associated gene repertoires and a CF-centered framework relating these repertoires to microbial phylogeny, genome-scale functional profiles, and infection-stage-associated variation in a murine *Toxoplasma gondii* infection model. CF-associated gene profiles can complement taxonomic and functional descriptions of the mouse gut microbiome, although the biological roles of individual CF-associated genes require experimental validation.

**IMPORTANCE:**

Studies of the mouse gut microbiome commonly emphasize taxonomic composition, whereas the ecological traits associated with microbial establishment and persistence in the murine intestine remain less well characterized. By profiling CF-associated gene repertoires across control, acute, and chronic infection groups in mice, we show that infection-stage-associated differences in murine gut microbial community structure are accompanied by differences in colonization- and fitness-associated gene profiles. CF composition was associated with microbial community structure and functional potential, and CF-based features captured intestinal-region- and infection-stage-associated variation within this mouse data set. The principal contribution of this study is a reference-based, CF-centered framework that complements conventional taxonomic and functional profiling in murine infection models and provides a foundation for future mechanistic investigations of microbial colonization.

## INTRODUCTION

The gut microbial community is a complex assemblage that plays a fundamental role in host health, disease, and development (1). These microorganisms contribute to essential aspects of host physiology (2), including nutrient metabolism (3), immune cell maturation (4), and resistance to pathogen colonization (5, 6). A critical determinant of microbial persistence in this environment is the ability of microorganisms to establish and maintain colonization within the intestinal niche. This process is mediated by a diverse array of genetic traits that enable adhesion, resource acquisition, and adaptation to host-associated conditions (7). In this study, colonization factors (CFs) refer to homology-inferred CF-associated gene families that include classical colonization-related determinants as well as broader genes associated with microbial persistence, host interaction, metabolic adaptation, and ecological fitness in the gut environment. Thus, CF-associated homologs identified here should be interpreted as components of a colonization- and gut fitness-associated gene repertoire, rather than as direct experimental evidence of colonization activity.

Emerging evidence from genome-resolved and phylogenetic analyses indicates that many microbial functional traits, including those associated with colonization, are non-randomly distributed across lineages, suggesting that evolutionary history constrains functional repertoires (8). This relationship is particularly evident for complex traits encoded by multiple genes, such as adhesion systems, biofilm formation, and host–microbe interaction pathways, which tend to be phylogenetically conserved (7, 8). Genome-resolved analyses have also revealed coordinated taxonomic succession and functional transitions across host developmental stages, including changes in chicken gut archaeal communities throughout the broiler growth cycle (9). More generally, closely related microorganisms often share similar functional repertoires and may occupy comparable ecological niches within the gut. In contrast, simpler traits, such as the utilization of specific substrates, are more phylogenetically dispersed due to frequent gain, loss, or horizontal gene transfer (8, 10). Despite these advances, how colonization-related traits are organized across microbial lineages, particularly at the level of specific CF repertoires, remains insufficiently understood.

In parallel, gut microbial community structure is known to be strongly shaped by host genetics (11), diet (12), inflammation (13), and disease state, with substantial shifts in taxonomic composition and diversity observed under perturbations such as infection (14, 15). However, most studies have focused on broad functional categories or metabolic pathways, leaving the specific organization, diversity, and ecological behavior of CFs across gut microbiota largely unresolved. This gap is particularly important in the context of infection, where microbial communities undergo coordinated structural and functional remodeling. Parasite-associated functional remodeling has also been reported in wild rodents infected with *Enterocytozoon bieneusi*, in which gut microbial pathways related to vitamins B and K2 biosynthesis were enhanced (16). For instance, *Toxoplasma gondii* infection has been associated with altered gut microbial composition and host metabolism in rats (17), while recent metabolomic profiling in mice has revealed extensive metabolic remodeling across acute and chronic infection stages (18). Stage-specific host responses are also evident beyond the intestine, as whole-transcriptome profiling of mouse brain revealed distinct regulatory networks during acute and chronic *T. gondii* infection (19). The acute and chronic phases of *T. gondii* infection represent biologically distinct host–parasite states characterized by differences in parasite developmental forms, tissue persistence, and host responses (20). Yet, it remains unclear whether CF-associated traits are systematically altered during infection, and how such changes relate to broader community dynamics and functional reorganization.

Addressing these questions requires a systematic characterization of CF diversity and distribution across microbial taxa, as well as an understanding of how these traits are structured phylogenetically and respond to environmental perturbations. In the current study, we integrate publicly available genomic, protein, and metagenomic datasets to perform a multi-layered analysis of CF repertoires across the mouse gut microbiome. We construct a comprehensive atlas of CF-associated gene diversity, evaluate the phylogeny-associated organization of CF-associated gene repertoires across microbial species, and examine their relationship with genome-scale functional profiles. In addition, we compare CF-associated gene profiles across intestinal regions and stages of *T. gondii* infection and assess their capacity to discriminate infection-associated microbiome states. By relating CF-associated gene diversity to microbial phylogeny, genome-scale functional profiles, and infection-stage-associated community variation, this work provides a computational framework for describing the organization of colonization- and fitness-associated traits in the murine gut microbiome.

## RESULTS

### A conserved yet expansive repertoire of CFs in the mouse gut microbiome

**Fig 1:**
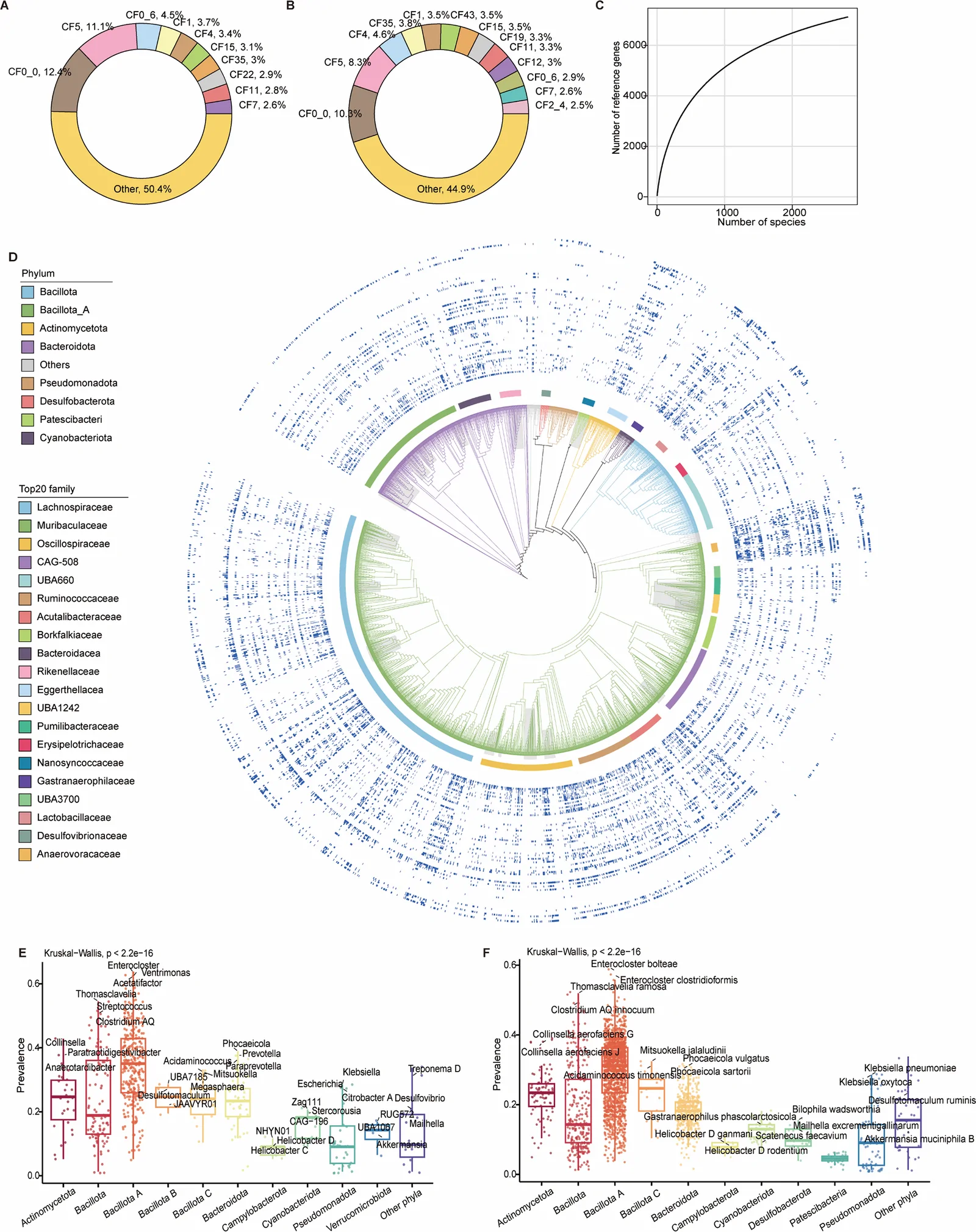
Global landscape and taxonomic distribution of colonization factor repertoires in the mouse gut microbiome. (**A and B**) Doughnut plots showing the composition of CF gene homologs identified in MGPC-90 (**A**) and after mapping to MGGC (**B**). (**C**) Species-level accumulation curve showing the number of reference CF genes detected as additional microbial species were sampled. (**D**) Phylogenetic tree of 2,824 mouse gut microbial species. Branches are colored by phylum-level taxonomy. Gray-highlighted clades indicate the 20 most species-rich genera; the middle ring denotes the 20 most species-rich families; the outer ring indicates the presence (blue) or absence (white) of CFs across species. (**E and F**) Boxplots showing CF prevalence at the genus level (**E**) and species level (**F**), grouped by phylum.

Motivated by a large-scale cross-species analysis of genetic determinants of gut microbial colonization (21), the landscape of CF-associated gene repertoires in the mouse gut microbiome was systematically characterized by integrating the Mouse Gut Protein Catalogue clustered at 90% protein identity (MGPC-90) and Mouse Gut Genome Catalogue (MGGC) (Table S1). Using sequence similarity searches, 53,148 protein-coding genes in MGPC-90 were matched to 7,127 reference CF genes, which grouped into 79 distinct CF families (Fig. 1A). Mapping these CFs onto the MGGC data set identified 2,406,371 CF gene homologs across 112,951 genomes representing 2,824 species (Fig. 1B). CF0_0 (Protein families database [Pfam]: *metE*) and CF5 (*metE*) showed the highest prevalence, each corresponding to large *metE*-like homologous repertoires within the reference database. Because *metE* is a conserved metabolic gene, these dominant families were interpreted as metabolic and gut fitness-associated components of the CF-associated repertoire rather than direct indicators of host-colonization activity. Rarefaction analysis indicated that the accumulation curves did not reach saturation (Fig. 1C).

To assess taxonomic distribution, a species-level CF presence/absence matrix was constructed and subsequently aggregated to higher taxonomic ranks. At the phylum level, Bacillota_A, Bacillota, Actinomycetota, and Bacteroidota contained the largest CF repertoires. Sixteen CFs were detected in more than 70% of phylum-level groups, including CF1, CF43, CF0_0, CF17, CF11, CF7, CF5, CF38, CF31, CF27, CF30, CF2_7, CF15, CF53, CF35, and CF37 (Fig. 1D). At finer taxonomic resolution, CF profiles differed significantly across genera and species (Kruskal–Wallis test, *P* <2.2 × 10^−16^; Fig. 1E and F). Within Bacillota_A, CF prevalence was highest among all phyla examined, while Actinomycetota and Bacteroidota also maintained relatively broad CF repertoires. Genera, including *Enterocloster*, *Acetatifactor*, *Blautia_A*, *Thomasclavelia*, and *Catenibacterium*, as well as species such as *Enterocloster bolteae*, *Enterocloster clostridioformis*, *Enterocloster aldenensis*, *Thomasclavelia ramosa*, and *Collinsella aerofaciens*, exhibited broader CF repertoires compared with many other lineages.

### Taxonomy-associated distribution and functional diversification of CF repertoires in the gut microbiome

**Fig 2:**
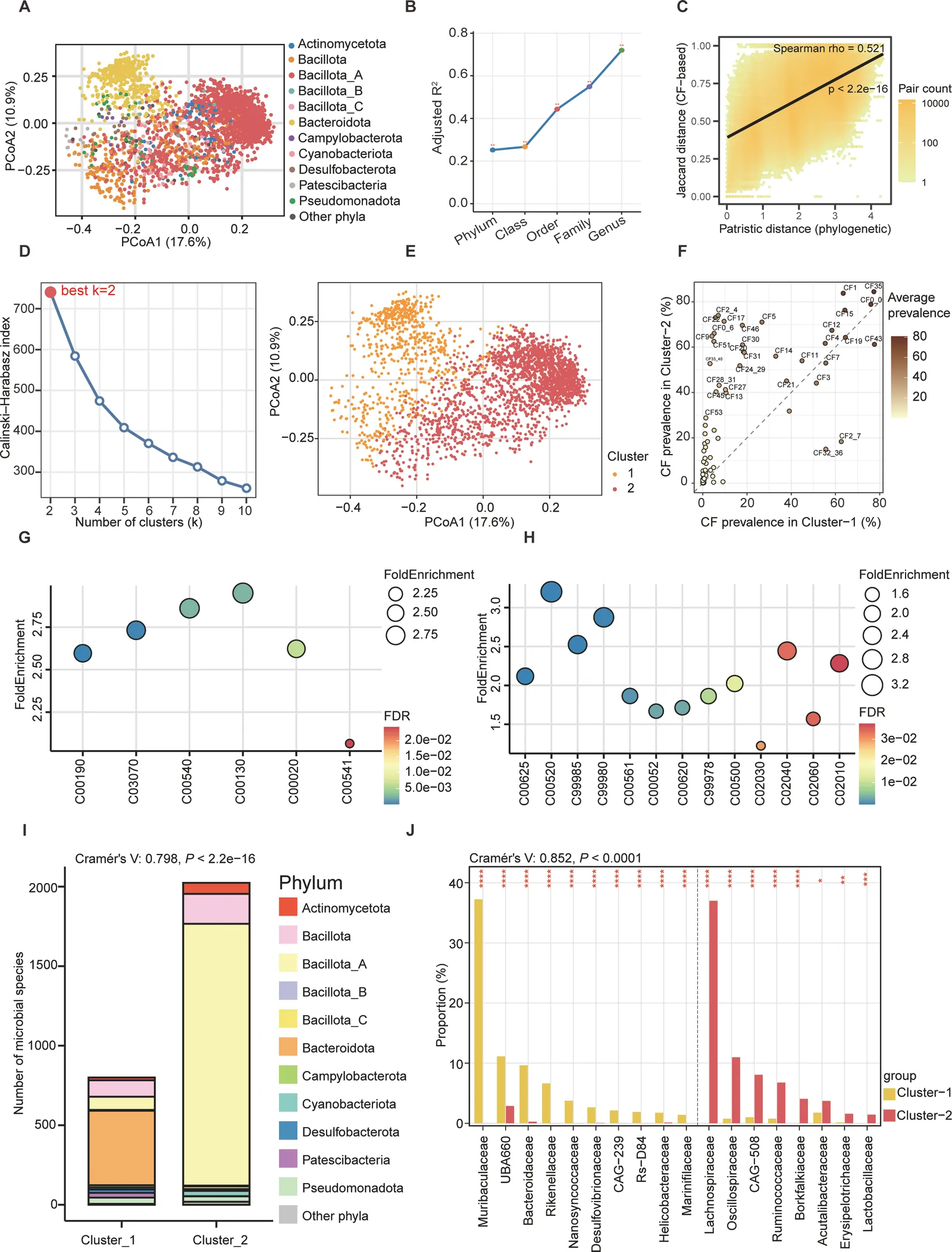
Phylogenetic structuring and functional differentiation of colonization factor repertoires across microbial lineages. (**A**) Principal coordinates analysis plot of species-level CF profiles based on Jaccard distance. Points represent species, positioned by PCoA1 and PCoA2, and colored by phylum. (**B**) Line plot showing the proportion of CF-profile variation explained by taxonomic rank (adjusted *R*^2^). Significance was assessed using PERMANOVA (adonis2, 999 permutations). (**C**) Hexbin plot showing the relationship between pairwise patristic distances and CF-based Jaccard distances. Color intensity indicates the number of species pairs per bin. Association was assessed using Spearman’s rank correlation. (**D**) Line plot showing the selection of the optimal number of clusters (*k*) for Partitioning Around Medoids (PAM) clustering based on the Calinski–Harabasz index. (**E**) PCoA plot showing PAM clustering results based on species-level CF profiles. (**F**) Scatter plot comparing CF prevalence between Cluster-1 and Cluster-2. Each point represents one CF; color indicates mean prevalence across clusters. (**G and H**) Bubble plots showing the top enriched Kyoto Encyclopedia of Genes and Genomes C-level pathways for Cluster-1-enriched (**G**) and Cluster-2-enriched (**H**) KOs. Bubble size represents fold enrichment; color indicates FDR. (**I**) Stacked bar plot showing the phylum-level distribution of species across the two clusters. (**J**) Bar plot showing family-level species distributions across cluster-associated families. Statistical significance in panels **I and J** was assessed using Fisher’s exact test with Benjamini–Hochberg correction (*FDR < 0.05, **FDR < 0.01, ***FDR < 0.001, and ****FDR < 0.0001).

To assess the relationship between CF composition and microbial phylogeny, principal coordinates analysis (PCoA) was performed using Jaccard distances derived from species-level CF profiles. Species were projected into a two-dimensional space and annotated by phylum-level taxonomy. A structured distribution was observed, with Bacillota_A forming a relatively compact group on the positive side of PCoA1, while Bacteroidota and Bacillota were mainly distributed on the negative side of PCoA1 and further separated along PCoA2. This pattern indicates a phylogeny-associated distribution of CF composition (Fig. 2A).

To quantify this relationship, PERMANOVA was applied across multiple taxonomic ranks. Taxonomy explained an increasing proportion of variation in CF profiles from higher to lower ranks, with adjusted *R*^2^ values of ~0.25 (phylum), 0.27 (class), 0.44 (order), 0.55 (family), and 0.72 (genus). All taxonomic levels were statistically significant (Fig. 2B). These results indicate increasing similarity in CF profiles with closer taxonomic relatedness.

At the species level, patristic distances were calculated and compared with CF-based Jaccard distances across 2,822 bacterial species. Spearman correlation analysis showed a positive association between phylogenetic distance and CF dissimilarity across all species pairs (Spearman’s rho = 0.521, *P* <2.2 × 10^−16^). This relationship remained consistent across 20 random subsamplings, with a median Spearman correlation of 0.52 and a range of 0.46–0.58. To determine whether this association exceeded the phylogenetic structure expected from general genome-content conservation, a prevalence-matched random KO gene-set null model was constructed. Using the same fixed set of 50,000 species pairs, the observed CF correlation was 0.524, whereas the median correlation across 999 matched random KO sets was 0.606. The CF correlation did not exceed the null expectation (empirical one-sided *P* = 0.898; Fig. 2C; Fig. S1 and S5). Thus, CF dissimilarity was positively associated with phylogenetic distance, and this relationship was embedded within the broader lineage-associated structuring of genome content.

Given this phylogeny-associated structure, species-level CF profiles were clustered using Partitioning Around Medoids (PAM) based on Jaccard dissimilarity. The Calinski–Harabasz index supported a two-cluster solution, whereas average silhouette width was slightly higher at *k* = 3, indicating that the two candidate solutions captured related and partially overlapping repertoire structures (Fig. 2D; Fig. S6A). As an independent clustering approach, divisive hierarchical clustering analysis (DIANA) showed greater internal support for *k* = 2 than for *k* = 3, with mean silhouette widths of 0.094 and 0.076, respectively (Fig. S6B). Agreement between PAM and DIANA was also greater at *k* = 2 than at *k* = 3 (adjusted Rand index = 0.447 versus 0.313). The DIANA solution at *k* = 2 contained two broad groups of 410 and 2,414 species, whereas the *k* = 3 solution introduced a small third group of 15 species. Considering these complementary results, the PAM solution at *k* = 2 was retained as a parsimonious representation of two broad CF repertoire patterns (Fig. S6B; Tables S2 and S7). Comparison of CF prevalence between clusters revealed distinct distribution patterns (Fig. 2F; Table S3). CF43 showed higher prevalence in Cluster-1, while CF1 and CF35 were more prevalent in Cluster-2. These CFs displayed asymmetric distribution between clusters, indicating differential representation across the two groups.

To evaluate whether CF-based clustering reflected broader functional differences, KO- and CF-based Jaccard distance matrices were compared across 2,824 shared species. Mantel’s test showed a significant correlation between the two matrices (Mantel’s *r* = 0.747, *P* = 0.001), indicating concordance between CF-associated gene composition and genome-wide functional gene profiles.

Differentially prevalent KOs between clusters were identified using Fisher’s exact test with FDR correction and fold-change filtering, followed by Kyoto Encyclopedia of Genes and Genomes (KEGG) pathway enrichment analysis. Cluster-1-enriched KOs included oxidative phosphorylation (C00190), citrate cycle (TCA cycle; C00020), ubiquinone and other terpenoid-quinone biosynthesis (C00130), lipopolysaccharide biosynthesis (C00540), biosynthesis of nucleotide sugars (C00541), and bacterial secretion system (C03070) (Fig. 2G; Table S4). Cluster-2-enriched KOs included ATP-binding cassette (ABC) transporters (C02010), phosphotransferase system (C02060), starch and sucrose metabolism (C00500), galactose metabolism (C00052), amino sugar and nucleotide sugar metabolism (C00520), bacterial chemotaxis (C02030), and flagellar assembly (C02040) (Fig. 2H; Table S4). Additional enriched categories in Cluster-2 included pyruvate metabolism (C00620), glycerolipid metabolism (C00561), cell growth (C99978), amino acid metabolism (C99985), enzyme functions (C99980), and chloroalkane and chloroalkene degradation (C00625).

Taxonomic mapping showed significant associations between CF-defined clusters and taxonomic composition at both the phylum (Cramér’s *V* = 0.798) and family (Cramér’s *V* = 0.852) levels (Fisher’s exact test, *P* <2.2 × 10^−16^ and *P* < 0.0001, respectively; Fig. 2I and J; Tables S5 and S6). Bacillota_A species were predominantly assigned to Cluster-2, whereas Bacteroidota were more represented in Cluster-1. Bacillota and Actinomycetota also contributed substantially to Cluster-2. At the family level, 18 cluster-associated families were retained for visualization. Muribaculaceae, UBA660, Bacteroidaceae, Rikenellaceae, and several additional families were enriched in Cluster-1, whereas Lachnospiraceae, Oscillospiraceae, CAG-508, Ruminococcaceae, Borkfalkiaceae, Acutalibacteraceae, Erysipelotrichaceae, and Lactobacillaceae were enriched in Cluster-2.

The PAM clusters were derived from species-level CF presence/absence profiles using Jaccard dissimilarity and therefore primarily captured variation in CF-associated gene composition. Taxonomic labels and KO profiles were incorporated only in subsequent comparative analyses. The significant associations with phylum- and family-level taxonomy, together with the concordance between CF- and KO-based distance matrices and the cluster-associated KO enrichments, showed that these CF repertoire patterns were closely linked to lineage-associated genomic backgrounds and broader predicted functional potential. Collectively, the two clusters represent broad CF repertoire configurations associated with corresponding taxonomic and functional genomic contexts. Finally, we assessed whether the principal species-level findings were robust to alternative CF-identification thresholds. When the query and subject coverage cutoffs were varied from 50% to 70%, the correlations between phylogenetic distance and CF dissimilarity remained similar, with Spearman’s rho values of 0.534, 0.527, and 0.521 at the 50%, 60%, and 70% thresholds, respectively. Varying the species-prevalence cutoff from 30% to 70% yielded corresponding correlations of 0.522, 0.521, and 0.477, respectively, all of which remained significant (*P* <2.2 × 10^−16^). The major taxonomic structure in the PCoA, the progressive increase in taxonomic explanatory power from phylum to genus, and the selection of *k* = 2 by the Calinski–Harabasz criterion were retained across the tested settings. Thus, reasonable variation in the coverage and species-prevalence thresholds affected the magnitude of some estimates but did not alter the direction or principal organization of the tested species-level findings (Fig. S3 and S4).

### Infection-stage-associated differences in CF diversity and composition

**Fig 3:**
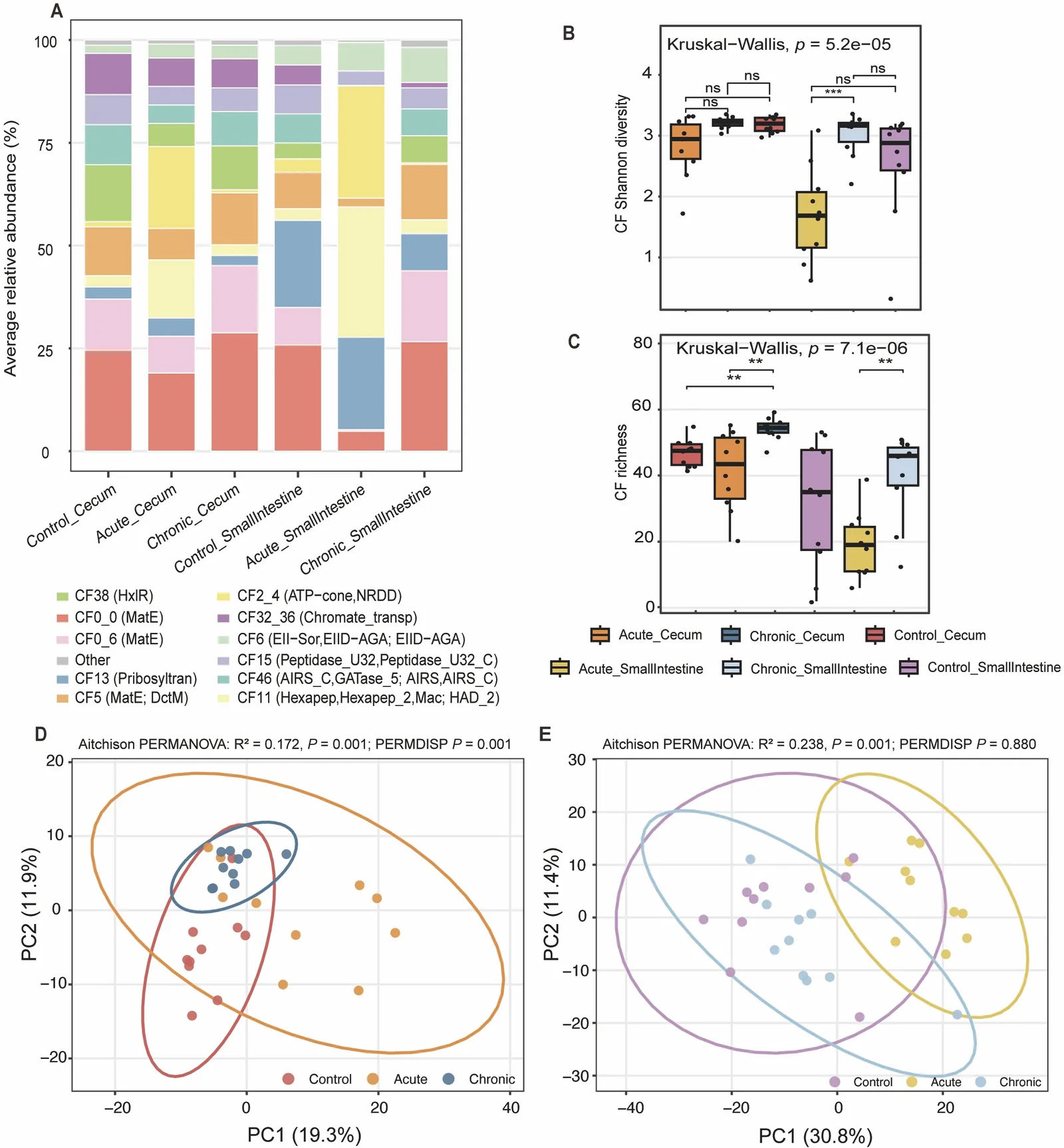
Infection-stage- and tissue-associated differences in colonization factor abundance, diversity, and composition. (**A**) Stacked bar plot showing the relative abundances of the top 11 CFs across six tissue–stage groups. Bars represent mean relative abundance; Pfam annotations are indicated in the legend. (**B and C**) Boxplots showing CF Shannon diversity (**B**) and CF richness (**C**) across tissue–stage groups. Statistical significance was assessed using the Kruskal–Wallis test followed by pairwise Wilcoxon rank-sum tests within each tissue. (**D and E**) Principal component analysis of CLR-transformed CF profiles in the cecum (**D**) and small intestine (**E**). Euclidean distances among CLR-transformed profiles were treated as Aitchison distances. Differences among infection groups were evaluated using PERMANOVA, and homogeneity of multivariate dispersion was assessed using PERMDISP with 999 permutations.

To assess infection-stage-associated differences in CF profiles, we analyzed cecal and small-intestinal samples from independent uninfected control, acute-infection, and chronic-infection groups, with 10 mice per group. Acute and chronic infection were defined as 11 and 33 days post-infection, respectively. Across all samples, CF0_0 showed the highest relative abundance, with a mean of 21.6% ± 8.9% (range: 4.8%–28.8%) across the six group–tissue combinations (Fig. 3A). This was followed by CF0_6 (10.8% ± 6.2%), CF13 (10.4% ± 9.2%), CF11 (9.6% ± 11.7%), CF5 (9.4% ± 4.2%), and CF2_4 (8.8% ± 11.7%). Relative abundances of these dominant CFs varied across both tissue type and infection stage. Descriptively, CF0_0 represented a smaller proportion of the CF profile in acute small-intestinal samples, whereas CF11 and CF2_4 represented larger proportions. These patterns highlight tissue- and infection-stage-associated shifts in the relative composition of dominant CFs.

CF alpha diversity was assessed using Shannon diversity and richness. Shannon diversity differed significantly across all groups after stratification by tissue and infection stage (Kruskal–Wallis test, *P* = 5.2 × 10^−5^). Pairwise comparisons showed that this difference was primarily driven by the small intestine, where the chronic group exhibited higher Shannon diversity than the acute group (Fig. 3B). CF richness also varied significantly across groups (Kruskal–Wallis test, *P* = 7.1 × 10^−6^). In the cecum, the chronic group showed higher richness than both the acute and control groups. In the small intestine, the chronic group showed higher richness than the acute group (Fig. 3C).

CLR-based PCA showed infection-stage-associated differentiation of CF profiles in both intestinal regions. Aitchison PERMANOVA identified significant differences among control, acute, and chronic groups in the cecum (*R*^2^ = 0.172, *P* = 0.001) and small intestine (*R*^2^ = 0.238, *P* = 0.001; Fig. 3D and E; Table S8).

Multivariate dispersion differed significantly among the cecal groups (PERMDISP, *F* = 12.434, *P* = 0.001; Fig. S7A and Table S8). The acute group showed the greatest mean distance to the group spatial median, the chronic group showed the lowest distance, and the control group was intermediate. Pairwise dispersion differences remained significant between the control and acute groups (Benjamini-Hochberg [BH]-adjusted *P* = 0.022), the control and chronic groups (BH-adjusted *P* = 0.022), and the acute and chronic groups (BH-adjusted *P* = 0.006). Accordingly, the significant cecal PERMANOVA result may reflect both differences in group location and differences in within-group heterogeneity.

In contrast, no significant difference in multivariate dispersion was detected among the small-intestinal groups (PERMDISP, *F* = 0.128, *P* = 0.899; Fig. S7B and Table S8). The small-intestinal result therefore supports infection-stage-associated differentiation of CF composition without a detectable contribution from unequal multivariate dispersion. Together, these results indicate tissue-dependent differences in the structure and heterogeneity of CF profiles across infection states.

### CF-associated gene profiles correspond with taxonomic community structure across intestinal regions and infection states

**Fig 4:**
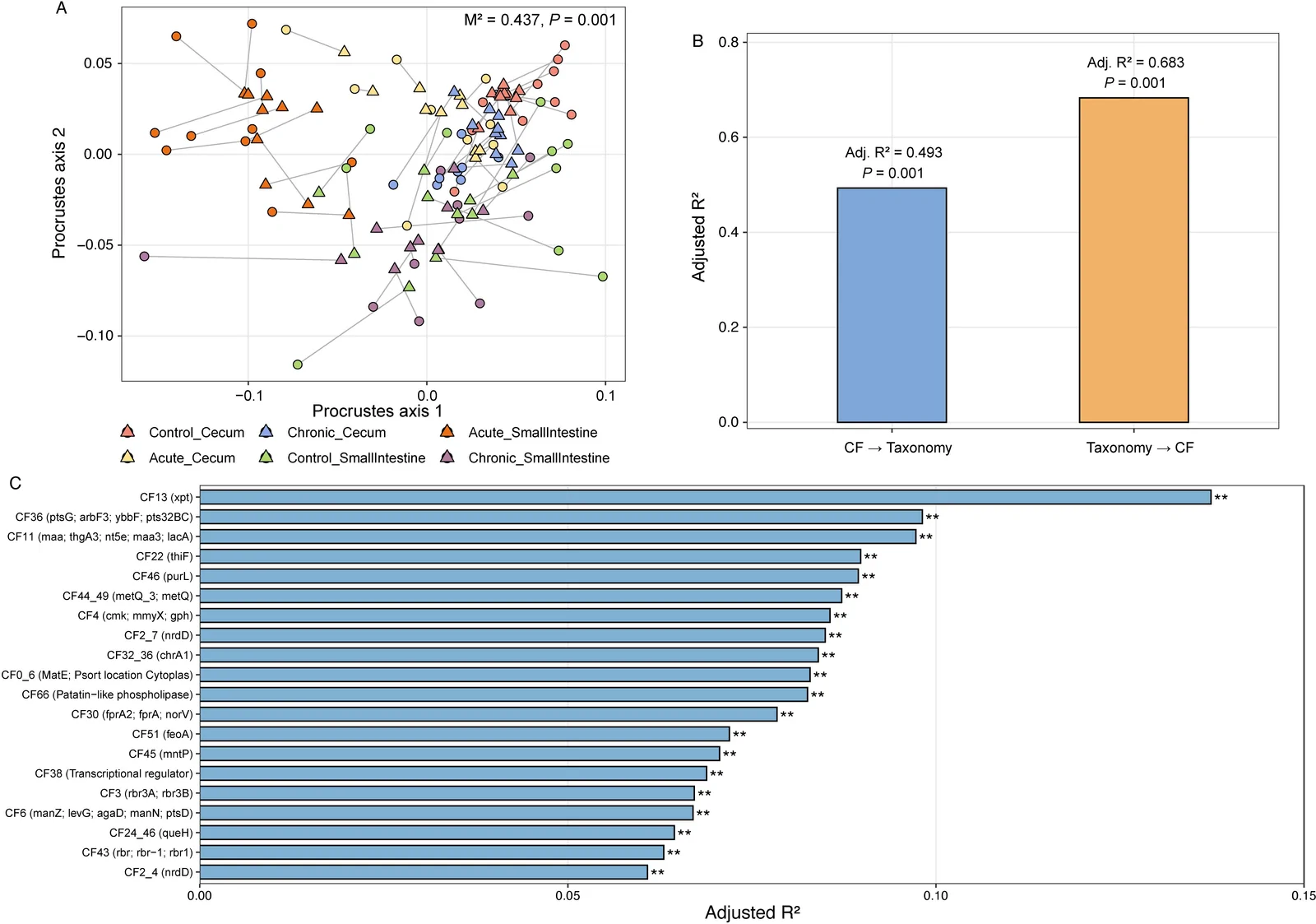
CLR-based concordance and statistical associations between colonization factor and taxonomic profiles. (**A**) Procrustes analysis comparing principal-component scores derived from CLR-transformed CF and species-level taxonomic profiles. Paired positions from the same sample are connected by gray lines, and significance was assessed using 999 permutations. (**B**) Reciprocal multivariate associations between CLR-transformed CF and taxonomic profiles. Adjusted *R*^2^ values were obtained from models using principal components accounting for at least 95% of the variance in the explanatory profile and Aitchison distances of the response profile. (**C**) Top 20 individual CLR-transformed CF features associated with species-level taxonomic Aitchison distances. Bars indicate adjusted *R*^2^ values, and *P* values were corrected using the Benjamini–Hochberg procedure.

Having established infection-stage-associated differences in CF composition, we next examined the correspondence between CF-associated gene profiles and species-level taxonomic community structure across the 60 metagenomic samples. Procrustes analysis yielded an *M*^2^ value of 0.437 and a Procrustes correlation of 0.751 (*P* = 0.001), indicating that the two profiles captured closely corresponding dimensions of microbiome variation across intestinal regions and infection states (Fig. 4A).

In the reciprocal multivariate analyses, principal components derived from CLR-transformed CF profiles were significantly associated with variation in species-level taxonomic Aitchison distances, with an adjusted *R*^2^ of 0.493 (*P* = 0.001). Taxonomic principal components were significantly associated with variation in CF Aitchison distances, with an adjusted *R*^2^ of 0.683 (*P* = 0.001; Fig. 4B). These results demonstrate a strong statistical correspondence between CF-associated gene profiles and taxonomic community structure after accounting for data compositionality.

Individual CLR-transformed CF features were also significantly associated with taxonomic community structure. Among the top 20 features, adjusted *R*^2^ values ranged from 0.061 to 0.137. Under the CLR/Aitchison framework, CF13, CF36, CF11, CF22, and CF46 showed the largest adjusted *R*^2^ values among the tested features. All top 20 features remained significant after Benjamini–Hochberg correction (adjusted *P* < 0.01; Fig. 4C; Table S8). Accordingly, interpretation focused primarily on the overall multivariate correspondence rather than on a fixed hierarchy of individual CF features. This correspondence provided a community-level context for the subsequent tissue- and infection-stage-specific differential abundance and classification analyses.

### Tissue- and infection-stage-associated CF differences and exploratory classification performance

To characterize tissue- and infection-stage-associated differences in CF profiles, differential abundance analysis was performed using DESeq2 across pairwise comparisons among acute, chronic, and control groups within each intestinal segment. Distinct CF abundance differences were observed among infection groups, with the largest number of differentially abundant CFs detected in comparisons involving the acute group, particularly in the small intestine (Fig. S2A, Table S9). In the small intestine, acute versus control and acute versus chronic comparisons showed widespread differences across multiple CFs, including decreases in many features and increases in a limited subset, such as CF2_4 and CF11. In contrast, chronic versus control comparisons identified fewer and more feature-specific differences. Cecal comparisons also involved fewer differentially abundant CFs than small-intestinal comparisons. Overall, the acute group differed most extensively from the other groups, whereas the chronic and control groups differed in a smaller subset of CF features.

Random forest classification was evaluated separately in 30 cecal and 30 small-intestinal samples using repeated stratified 10-fold cross-validation. In the cecum, the All-CF, species-level, and genus-level models achieved median macro-AUC values of 0.946, 0.938, and 0.977, respectively, with corresponding median accuracies of 0.833, 0.833, and 0.867. Thus, all three models showed high discriminatory performance, with the genus-level model yielding the highest median macro-AUC and accuracy. In the small intestine, the species- and genus-level taxonomic models showed higher median macro-AUC values of 0.902 and 0.905, respectively, than the All-CF model at 0.788. The corresponding median accuracies were 0.800 for the species-level model, 0.767 for the genus-level model, and 0.667 for the All-CF model. These results indicate that taxonomic profiles provided stronger discrimination in the small intestine, whereas CF profiles retained high discriminatory performance in the cecum (Fig. S2B and Table S10).

## DISCUSSION

This study provides a genome-scale characterization of CF genes in the mouse gut microbiome using large-scale genome and protein catalogs. In this work, CFs are interpreted as homology-inferred gene families associated with microbial establishment, persistence, host interaction, metabolic adaptation, and gut ecological fitness. This definition encompasses canonical colonization-related determinants as well as broader fitness-associated and metabolic components of the gut microbial gene repertoire (22, 23).

The mouse gut microbiome contained a large and highly structured repertoire of CF-associated genes spanning 79 families and more than 2.4 million homologs across over 2,800 microbial species. A small number of CF families, including CF0_0 (Pfam: *metE*) and CF5 (*metE*), dominated the overall landscape, indicating that some CF-associated genes are broadly conserved and frequently expanded across diverse microbial lineages. The dominance of *metE*-like families should therefore be interpreted cautiously. Rather than indicating direct colonization activity, their broad distribution may reflect conserved metabolic capacity, gut environmental fitness, or lineage-specific expansion of metabolic genes across mouse gut microbial genomes. Rarefaction analysis further suggested that CF diversity is not yet fully captured, implying that additional CF-associated functions are likely to be discovered as more genomes become available.

CF repertoires were not randomly distributed across bacterial lineages but showed strong taxonomic structure. Major gut-associated phyla, including Bacillota_A, Bacillota, Actinomycetota, and Bacteroidota, contained the largest CF repertoires, suggesting that these lineages carry broader CF-associated functional potential compared with other groups. At finer taxonomic resolution, CF profiles became increasingly lineage-specific, indicating a hierarchical organization in which broad conservation across phyla coexists with divergence at the genus and species levels. A subset of CF families was highly conserved, with 16 CF families present in more than 70% of phylum-level groups, supporting the existence of a core CF repertoire shared across deep evolutionary divisions.

These patterns indicate that CF-associated genes are both conserved and diversified across microbial lineages, consistent with broader lineage-associated variation in microbial genome content. CF composition showed a clear phylogeny-associated pattern across the mouse gut microbiome. Species-level CF profiles varied with taxonomic affiliation, and phylogenetic distance was positively associated with CF dissimilarity across repeated subsampling. The prevalence-matched random KO null model further showed that the magnitude of this association was within the range expected from general genome-content conservation. Together, these findings indicate that CF repertoires follow reproducible lineage-associated patterns embedded within the broader genomic similarity of related microbial taxa, while providing a trait-focused perspective on the distribution of colonization- and fitness-associated functions (24–26). The retention of the taxonomic organization, positive phylogenetic association, and two-group PAM structure across alternative alignment-coverage and species-prevalence thresholds further supports the robustness of these broad CF repertoire patterns.

CF-based clustering identified two broad repertoire patterns across microbial species. Although the candidate clustering solutions were not sharply separated, the two-cluster PAM solution provided a concise and biologically interpretable summary of the dominant variation in CF composition. This interpretation was supported by the Calinski–Harabasz index and by the greater internal separation and stronger agreement with PAM observed for DIANA at *k* = 2 than at *k* = 3. The resulting PAM groups were defined by variation in species-level CF presence/absence profiles and therefore represent broad and partially overlapping CF repertoire configurations. Their distinct CF prevalence profiles, taxonomic distributions, and KO-associated pathway enrichments further indicate that these configurations are linked to lineage-associated genomic backgrounds and broader differences in predicted functional potential.

Cluster-1 showed greater representation of pathways related to oxidative phosphorylation, the citrate cycle, quinone and nucleotide-sugar biosynthesis, lipopolysaccharide biosynthesis, and bacterial secretion. In contrast, Cluster-2 showed greater representation of ABC transporters, phosphotransferase systems, carbohydrate metabolism, bacterial chemotaxis, and flagellar assembly. These pathway-level differences characterize variation in predicted functional potential across the two CF-associated groups. In conjunction with the pronounced phylum- and family-level taxonomic structure of the clusters, the enrichment patterns likely reflect the combined contributions of CF repertoire differentiation and lineage-associated genomic characteristics.

Beyond these species-level repertoire patterns, *T. gondii* infection stage was associated with differences in CF profiles across intestinal regions. Although a subset of CFs remained dominant across all groups, their relative representation differed among tissue–stage groups, particularly in acute small-intestinal samples. CLR/Aitchison analyses further identified significant differences in overall CF composition in both intestinal regions. These findings indicate that infection states were associated with tissue-dependent differences in CF diversity and compositional structure.

Multivariate dispersion based on Aitchison distances differed among infection groups in the cecum. The acute group showed the greatest distance to the group spatial median, the chronic group showed the lowest distance, and the control group was intermediate. All three pairwise dispersion comparisons remained significant after Benjamini–Hochberg correction. Because PERMANOVA can be influenced by both differences in group location and within-group dispersion, the significant cecal result should be interpreted as overall multivariate differentiation rather than as evidence of group-centroid separation alone. In contrast, no significant difference in multivariate dispersion was detected among the small-intestinal groups, supporting a more direct interpretation of infection-group-associated compositional differentiation in this tissue.

In addition to these infection-stage-associated differences, the CLR/Aitchison analyses supported a strong statistical correspondence between CF-associated gene profiles and taxonomic community structure. Significant Procrustes concordance and reciprocal multivariate associations indicate that CF and taxonomic profiles capture closely related dimensions of microbiome variation across intestinal regions and infection states. At the individual-feature level, CF13, CF36, CF11, CF22, and CF46 showed the largest adjusted *R*^²^ values under the CLR/Aitchison framework, identifying a subset of CF features that were most closely associated with variation in taxonomic community structure.

Differential abundance analyses further showed tissue- and infection-stage-associated differences in CF profiles. The acute group showed the most extensive differential-abundance pattern, characterized by widespread CF depletion in the small intestine and more selective differences in the cecum, whereas chronic-versus-control comparisons involved fewer CF features. These results indicate that the acute group showed the most extensive CF-associated differences, whereas the chronic group differed from controls in a more limited subset of CF features.

Exploratory machine-learning analyses revealed tissue-dependent differences in the discriminatory information captured by CF and taxonomic profiles. In the cecum, all three feature sets achieved high classification performance, with the genus-level model showing the highest median macro-AUC and accuracy, while the All-CF model also retained strong discriminatory performance. In the small intestine, both species- and genus-level taxonomic profiles showed stronger performance than the All-CF model. These results suggest that CF profiles capture an informative trait-focused dimension of microbiome variation, whereas the relative contribution of CF and taxonomic representations to infection-stage discrimination differs across intestinal regions. Accordingly, CF-associated gene and taxonomic profiles provide complementary views of infection-associated microbiome variation.

Taken together, these findings position CF-associated gene profiles as an additional organizational layer within broader genome-scale patterns in the mouse gut microbiome. The main contribution of this study lies in integrating phylogenetic, functional, and infection-stage-associated relationships within a CF-centered computational framework. However, the homology-based, computational, and observational nature of the study precludes mechanistic or causal inference for individual CF-associated genes (24, 25). Nutrient- and niche-related factors (27–29), as well as intestinal inflammation, tissue injury, mucosal immune responses, and feeding-related changes (27, 30–32), may also influence bacterial colonization but were not directly measured. The cross-sectional design cannot resolve within-animal temporal trajectories; therefore, the reported CF patterns should be interpreted as features of integrated infection states rather than as parasite-specific CF remodeling. Experimental, longitudinal, and external validation will be required to establish the functions and generalizability of individual CF-associated features.

### Conclusions

This study provides a reference-based atlas of CF-associated gene repertoires in the mouse gut microbiome. Within the murine *T. gondii* infection model, CF-associated gene profiles showed phylogeny-, tissue-, and infection-stage-associated patterns and were statistically associated with taxonomic and genome-scale functional profiles. The principal contribution of this work is a CF-centered framework that complements conventional descriptions of the mouse gut microbiome. Experimental studies are required to determine whether individual CF-associated genes directly contribute to microbial colonization, persistence, or infection-associated community changes.

## MATERIALS AND METHODS

### Data curation

A comprehensive data set of CF gene sequences was assembled from multiple sources to ensure broad coverage and reproducibility. A total of 27,096 amino acid sequences corresponding to the CF reference genes, representing 79 CF families, were obtained from the genome–habitat association (GHA) project (https://tavazoielab.c2b2.columbia.edu/GHA/). This data set was selected because it provides a standardized and reproducible set of colonization-associated gene families derived from a large-scale comparative framework, making it suitable for systematic profiling across the MGPC-90 and MGGC resources. In this study, the GHA-derived 79 CF families were used as a core reference set for constructing a mouse gut CF-associated gene atlas. The resulting atlas therefore provides a reference-based framework for characterizing CF-associated gene repertoires in the mouse gut microbiome and can be expanded as additional experimentally supported CFs become available. Because the GHA-derived reference set is not exhaustive, recently characterized, strain-specific, host-specific, context-dependent, or poorly annotated colonization mechanisms may be underrepresented. To characterize CF gene representation in the mouse gut microbiome, two complementary catalogs were used: the MGGC and MGPC-90, version 1.0. These data sets comprise 112,951 nonredundant genomes and 8,038,712 nonredundant genes clustered at 90% protein identity. Both catalogs were downloaded from the European Bioinformatics Institute MGnify database (33) (https://ftp.ebi.ac.uk/pub/databases/). These resources were chosen because they provide large-scale, nonredundant representations of microbial genomes and proteins, enabling robust identification of CF genes in the mouse gut environment.

### Infection-associated metagenomic data set and experimental design

Mouse gut metagenomic sequencing data associated with *Toxoplasma gondii* infection were obtained from the NCBI BioProject database under accession number PRJNA1419542. The original experiment used a stage-resolved cross-sectional design involving 30 specific-pathogen-free female C57BL/6J mice aged 6–8 weeks. Mice were randomly assigned to an uninfected control group, an acute-infection group, and a chronic-infection group, with 10 independent animals per group.

The mice were housed in a pathogen-free facility equipped with an independent ventilation system at 22°C ± 2°C and 50% ± 10% relative humidity under a 12-h light/12-h dark cycle, with food and water provided *ad libitum*. All mice were acclimated for 1 week before the experiment.

The low-virulence PRU strain of *T. gondii* (type II), maintained in Kunming mice, was used for infection. Mice in the acute- and chronic-infection groups received 20 brain cysts by oral gavage, whereas mice in the control group received an equivalent volume of PBS. The uninfected control group was sampled at day 0, whereas acute and chronic infection were defined as 11 and 33 days post-infection, respectively. Cecal and small-intestinal samples were collected from each mouse, yielding 60 metagenomic samples in total, with 10 samples for each infection-stage-by-intestinal-region combination. The metagenomic libraries were sequenced to an approximate depth of 10 Gb per sample.

### Identification of conserved CF homologs in the mouse gut microbiome

In this study, CFs refer to homology-inferred CF-associated gene families identified based on sequence similarity to a curated reference data set of colonization-associated genes. This definition includes classical colonization-related determinants as well as broader genes associated with microbial persistence, host interaction, metabolic adaptation, and ecological fitness in the gut environment. The identified CF homologs provide a sequence similarity-based framework for characterizing colonization- and fitness-associated gene repertoires across the mouse gut microbiome and for prioritizing individual CF-associated families for future functional investigation. To identify conserved CF homologs in the mouse gut microbiome, a sequence similarity-based approach was used. This method enables sensitive and high-throughput detection of homologous proteins across large-scale metagenomic data sets. Protein sequences from MGPC-90 were aligned against a curated set of reference CF protein sequences using DIAMOND (34) (v2.1.8). Alignments were performed in “blastp” mode. For each query sequence, only the best-scoring hit was retained. A sequence was classified as a conserved CF homolog when the retained alignment met all of the following criteria: an *E*-value <1 × 10^−10^, amino acid identity >50%, query coverage >70%, and subject coverage >70%. Query and subject coverage were calculated as the aligned length divided by the full length of the corresponding query and subject sequences, respectively. These thresholds were used to retain well-aligned, high-confidence homologs for downstream comparative profiling. Representative CF protein sequences were functionally annotated using eggNOG-mapper (35) (v2.1.6, database v5.0.2).

Kyoto Encyclopedia of Genes and Genomes and Protein families database annotations were extracted to support downstream functional interpretation. These annotation sources were used because they provide complementary information on metabolic pathways and protein domains. To maintain a high-confidence and interpretable CF data set, CF40, CF58, and CF62 were excluded from subsequent functional annotation, species-level mapping, and phylogenetic analyses. CF-family annotations were first assigned to MGPC-90 representative protein clusters based on the retained DIAMOND matches. The cluster-membership relationships provided with MGPC were then used to propagate each CF-family assignment from the MGPC-90 representative sequence to all protein members assigned to the same 90%-identity cluster. Each member protein retained its source MGGC genome identifier, allowing the expanded CF homolog records to be linked to their corresponding genomes and species. Thus, the 53,148 CF-associated MGPC-90 representative proteins were expanded to 2,406,371 genome-associated homolog records across 112,951 genomes. Genome-level homolog records were retained when calculating the total number of CF homologs, whereas species-level CF presence was determined separately from the proportion of genomes carrying each CF family. A CF was considered present in a species only if it was detected in at least 50% of the genomes belonging to that species. These criteria were applied to distinguish sporadic detection from consistent presence within a species. To evaluate the sensitivity of the species-level CF profiles to the identification thresholds, one-factor-at-a-time sensitivity analyses were performed. Query and subject coverage cutoffs of 50%, 60%, and 70% were evaluated while maintaining the species-prevalence cutoff at 50%. The species-prevalence cutoff was separately evaluated at 30%, 50%, and 70% while maintaining both query and subject coverage cutoffs at 70%. For each threshold setting, the species-level CF presence/absence matrix was reconstructed, and Jaccard-based PCoA, taxonomic PERMANOVA, Spearman correlation between patristic and CF-based Jaccard distances, repeated stochastic subsampling, and PAM cluster selection using the Calinski–Harabasz index were repeated using the same analytical procedures. The 20 stochastic subsamplings used random seeds 2027–2046. A phylogenetic tree was constructed using PhyloPhlAn (36) (v3.1.68). This tool enables accurate reconstruction of microbial phylogenies from genome-scale data. The resulting tree was visualized using Interactive Tree of Life (v7.3, https://itol.embl.de/).

### Metagenome bioinformatic analysis

Raw sequencing reads from all samples were processed using fastp (37) (v0.23). Reads were removed if they met any of the following criteria: (i) contained more than 55 bases with quality scores below 20, (ii) included more than five ambiguous bases (“N”), or (iii) showed low sequence complexity or adapter contamination. These filters were applied to retain only high-quality reads suitable for downstream analysis. To eliminate host contamination, filtered reads were aligned to the mouse reference genome (GRCm39, GCF_000001635.27) using Bowtie2 (38) (v2.5). Reads that mapped to the mouse genome were discarded. This step ensured that subsequent analyses focused exclusively on microbial sequences. High-quality, non-host reads were aligned to the nucleotide sequences of CF-annotated genes to estimate their abundance. CF gene abundance was quantified as transcripts per kilobase per million mapped reads (TPM), accounting for both gene length and sequencing depth. TPM-normalized abundances were used for descriptive abundance profiling and compositional analyses, whereas the unnormalized raw integer read counts assigned to each CF family were retained separately for DESeq2 differential abundance analysis. Normalization and read processing were performed using Samtools (v1.21) together with in-house scripts (21). A CF gene was considered present in a sample only if read coverage across the gene exceeded 50%. This threshold was applied to reduce false-positive detections and ensure reliable identification of abundant CF genes.

### Gut bacteriome profiling

The MGGC database was used as the reference for taxonomic profiling. This database provides a comprehensive and nonredundant collection of mouse gut microbial genomes, allowing accurate species-level classification. Sequencing reads were taxonomically classified using Kraken2 (39) (v2.1.3). This tool assigns reads by matching k-mers to reference genomes, enabling efficient classification of large metagenomic data sets. Read count matrices spanning multiple taxonomic ranks, from phylum to species, were generated and standardized using Bracken (v3.0.1). Bracken was used to refine abundance estimates from Kraken2 outputs, improving the accuracy of taxonomic quantification across samples.

### Cluster and correlation analysis

Patristic distances among 2,822 bacterial species were extracted from the phylogenetic tree and compared with Jaccard dissimilarities calculated from the species-level CF presence/absence matrix using Spearman’s rank correlation. Robustness was evaluated using 20 random subsamplings, each retaining 0.01% of all pairwise species comparisons. To evaluate whether the observed CF–phylogeny association exceeded that expected from general genome-content conservation, a prevalence-matched random KO gene-set null model was constructed. KOs were selected from a background set of 7,762 KOs and matched to the retained CF families according to their species prevalence. A total of 999 random KO sets were thus generated. Jaccard dissimilarities and their Spearman correlations with patristic distance were calculated for each random set using the same fixed 50,000 species pairs. The one-sided empirical *P* value was calculated as the proportion of null correlations equal to or greater than the observed CF correlation. The fixed set of 50,000 species pairs was sampled using random seed 2026, and the 999 prevalence-matched random KO sets were generated using iteration-specific seeds 10001–10999.

PAM clustering was applied to species-level CF presence/absence profiles using Jaccard dissimilarity. Candidate cluster numbers from *k* = 2 to *k* = 10 were evaluated using the Calinski–Harabasz index and average silhouette width. Divisive hierarchical clustering analysis was additionally performed using the same Jaccard dissimilarity matrix as an independent clustering approach. Because *k* = 2 and *k* = 3 represented the leading candidate solutions identified by the PAM-based validation criteria, the corresponding DIANA partitions were further evaluated using mean silhouette width, cluster-size distribution, and agreement with the PAM assignments. Agreement between clustering methods was quantified using the adjusted Rand index. To identify CFs associated with each cluster, the prevalence of each CF was calculated separately within Cluster-1 and Cluster-2. Differences in prevalence between clusters were assessed using Fisher’s exact test. Enrichment fold change was defined as the ratio of CF prevalence within one cluster relative to the other. *P* values were adjusted for multiple testing using the Benjamini-Hochberg procedure. To examine functional differences between clusters, a binary matrix of KEGG Ortholog (KO) presence by genome was constructed from genome-scale KO annotations. The analysis was restricted to samples shared with the CF data set to ensure comparability.

Correlation between KO-based and CF-based Jaccard distance matrices was assessed using Mantel’s test with Spearman correlation, implemented in the vegan package (999 permutations). This test was used to evaluate the association between CF composition and functional gene content. Differentially prevalent KOs between the two clusters were identified using Fisher’s exact test on 2 × 2 contingency tables with simulated *P*-values, followed by BH correction. KOs with adjusted *P*-values <0.05 and enrichment fold change >2 were considered cluster-enriched. KEGG pathway enrichment analysis of cluster-enriched KOs was performed using clusterProfiler, based on the official KO BRITE hierarchy. For visualization, only KEGG C-level pathway terms were retained. BRITE-like, unknown, prediction-related, or otherwise non-standard labels were excluded prior to plotting to improve interpretability.

### Rarefaction curve and diversity analyses

Species-level accumulation curves were generated using the specaccum function in the vegan package to evaluate how the number of detected reference CF genes increased with the number of microbial species sampled and whether the catalog-level CF repertoire approached saturation. Alpha diversity indices, including Shannon diversity and richness, were calculated from sample-level CF abundance profiles.

### Compositional analysis of CF and taxonomic abundance profiles

Relative-abundance profiles were calculated for descriptive visualization of dominant CF distributions across samples. Inferential analyses of multivariate CF and taxonomic abundance profiles were performed within a compositional data framework.

Within each analysis set, features with nonzero abundance in at least 20% of samples were retained to reduce instability associated with extremely sparse features. This corresponded to a minimum prevalence of 6 of 30 samples in each tissue-specific CF analysis and 12 of 60 samples in the combined CF–taxonomy analysis. Zero values were replaced using count-zero multiplicative replacement, as implemented in the zCompositions package, followed by centered log-ratio transformation. Euclidean distances calculated from the CLR-transformed profiles were treated as Aitchison distances.

Tissue-specific CF composition was visualized using principal component analysis of CLR-transformed profiles. Differences among control, acute, and chronic groups were evaluated using PERMANOVA based on Aitchison distances with 999 permutations. Homogeneity of multivariate dispersion was assessed using PERMDISP with bias-adjusted distances to the spatial median and 999 permutations. When the overall PERMDISP test was significant, pairwise comparisons were performed, and the resulting *P* values were adjusted using the Benjamini–Hochberg procedure.

To evaluate the correspondence between CF and taxonomic profiles, PCA was performed separately on the CLR-transformed CF and species-level taxonomic matrices. Procrustes analysis was conducted using an equal number of principal-component axes from the two data sets. The number of retained axes was defined as the smaller of the numbers required to account for at least 95% of the variance in each data set. Statistical significance was evaluated using 999 permutations.

Reciprocal multivariate associations were evaluated using principal components accounting for at least 95% of the variance in the explanatory profile and the Aitchison distance matrix of the response profile. Individual CLR-transformed CF features were separately tested for association with species-level taxonomic Aitchison distances using PERMANOVA with 999 permutations. *P* values from the individual-CF analyses were adjusted using the Benjamini–Hochberg procedure. The CLR/Aitchison workflow was initialized using random seed 2025. The overall cecal and small-intestinal PERMDISP tests used seeds 2025 and 2026, respectively, and the cecal pairwise PERMDISP comparisons used seeds 2031–2033. The permutation-based CF–taxonomy correspondence analyses were performed in a fixed script order after initializing the random-number generator with seed 2025.

### Pairwise differential analysis of CF features

Differential analysis was conducted using DESeq2 based on CF-level read-count matrices (40). Pairwise comparisons were performed among acute, chronic, and control groups within each intestinal segment. *P* values were adjusted using the Benjamini–Hochberg procedure to control the false discovery rate.

### Random forest-based machine learning approach

Random forest classification was performed separately for the cecum and small intestine following established microbiome machine-learning procedures (41). Each tissue-specific data set contained 30 balanced samples, including 10 acute, 10 chronic, and 10 control samples. Three feature sets were evaluated: CF abundance profiles, species-level taxonomic profiles, and genus-level taxonomic profiles.

Model performance was assessed using stratified 10-fold cross-validation repeated 100 times, with identical fold assignments across feature sets. A base random seed of 2025 was used to ensure reproducibility. For repetition *r*, stratified fold assignment used seed 2025 + *r*. Random forest fitting for feature set *m* and fold *f* used seed 2025 + 1000*r* + 100000*m* + *f*, where *m* = 1, 2, and 3 represented the All-CF, species-level, and genus-level feature sets, respectively, and *f* = 1–10 represented the cross-validation fold. Zero-variance features were removed using the training data within each fold, and random forest models were fitted with 1,000 trees. Out-of-fold probabilities were used to calculate class-specific AUC, macro-AUC, and accuracy, which were summarized as medians and 2.5^th^–97.5^th^ percentile empirical intervals. Before within-fold filtering, the input matrices contained 66 CF features, 2,565 species-level taxonomic features, and 565 genus-level taxonomic features.

### Statistical analyses and visualization

Large-scale sequence-similarity searches, genome-level mapping, and phylogenetic analyses were performed on a Linux computing server. Downstream statistical analyses and visualization were conducted in R v4.4.3. Software versions and relevant non-default parameters are reported in the corresponding methodological subsections; parameters not otherwise specified were retained at their default settings. A combination of multivariate association tests, differential abundance methods, and non-parametric statistics was used to ensure robust assessment of relationships between CF profiles, taxonomic structure, and experimental groups. To evaluate the relationship between taxonomic distribution and PAM cluster assignments, Cramér’s V statistic was calculated using rstatix (v0.7.2). This measure was used to quantify the strength of association between categorical variables. At finer taxonomic resolution, associations between taxonomic categories and PAM clusters were assessed at both phylum and family levels. For each phylum or family, enrichment in one cluster relative to the other was tested using Fisher’s exact test applied to 2 × 2 contingency tables. Simulated *P*-values (*B* = 10,000) were used to improve estimation accuracy, and the resulting *P*-values were adjusted using the BH procedure to control for multiple testing.

Differential abundance analysis of CF features was performed using DESeq2 (v1.42.1) with unnormalized raw integer CF read counts as input. Pairwise comparisons were conducted among the acute, chronic, and control groups separately within each intestinal region. DESeq2 normalization based on internally estimated size factors was applied to account for differences in sequencing depth, and differential abundance was evaluated using the negative binomial modeling framework implemented in DESeq2. Enrichment analysis of differentially prevalent KOs was performed using clusterProfiler based on the official KO BRITE hierarchy (https://www.kegg.jp/brite/ko00001). Only KOs meeting the criteria of FDR <0.05 and enrichment fold change >2 were included in enrichment analyses to ensure biological relevance and statistical confidence. For group comparisons involving non-normally distributed data, the Kruskal–Wallis test was used for multi-group analyses, followed by pairwise Wilcoxon rank-sum tests where appropriate. Ternary plots were generated using ggtern (v3.5). General data visualization was performed using ggplot2 (v3.5.2) and ggpubr (v0.6.1), enabling consistent and publication-quality graphical outputs.

## Data Availability

The MGGC and MGPC-90 resources used in this study are publicly available through the EBI MGnify database. The infection-associated mouse gut metagenomic sequencing data are available from the NCBI Sequence Read Archive under BioProject accession PRJNA1419542. The R analysis scripts and accompanying run.sh file used in this study are publicly available in Figshare at https://doi.org/10.6084/m9.figshare.33005537.

## SUPPLEMENTAL MATERIAL

Figure S1 spectrum.01940-26-s0001.tif Robustness of the association between phylogenetic distance and CF compositional dissimilarity.

Figure S2 spectrum.01940-26-s0002.tif Differential abundance of CFs and repeated cross-validation performance of CF- and taxonomy-based classifiers across intestinal regions.

Figure S3 spectrum.01940-26-s0003.tif Sensitivity of species-level CF repertoire analyses to alternative sequence-coverage thresholds. Species-level CF presence/absence matrices were reconstructed using relaxed paired query/subject coverage cutoffs, while the species-prevalence cutoff was fixed at 50%.

Figure S4 spectrum.01940-26-s0004.tif Sensitivity of species-level CF repertoire analyses to alternative species-prevalence thresholds. Species-level CF presence/absence matrices were reconstructed using alternative species-prevalence cutoffs, while the paired query/subject coverage cutoffs were fixed at 70/70.

Figure S5 spectrum.01940-26-s0005.tif Prevalence-matched random KO gene-set null model for the association between phylogenetic distance and gene-content dissimilarity.

Figure S6 spectrum.01940-26-s0006.tif Complementary validation of species-level CF repertoire clustering.

Figure S7 spectrum.01940-26-s0007.tif Multivariate dispersion of CLR-transformed CF profiles across infection groups in the cecum and small intestine.

Supplemental legends spectrum.01940-26-s0008.docx Legends for Fig. S1 to S7.

Tables S1 to S10 spectrum.01940-26-s0009.xlsx Functional annotation, clustering results, taxonomic and functional enrichment analyses, compositional analyses, differential abundance results, and repeated cross-validation performance.

ASM does not own the copyrights to Supplemental Material that may be linked to, or accessed through, an article. The authors have granted ASM a non-exclusive, world-wide license to publish the Supplemental Material files. Please contact the corresponding author directly for reuse.
