# Supplementary material for: An atlas of colonization factors in the mouse gut microbiome reveals phylogenetically structured repertoires and infection-stage-associated variation during Toxoplasma gondii infection in mice
Source: Microbiol Spectr. 2026 Sep 22;14(11):e01940-26. doi: 10.1128/spectrum.01940-26 (PMC13729138; doi:10.1128/spectrum.01940-26)
Supplement: Supplementary file 1 [file spectrum.01940-26-s0008.docx]

**Figure S1. Robustness of the association between phylogenetic distance and CF repertoire dissimilarity.** Scatter plots show the relationships between pairwise patristic distances and CF-based Jaccard dissimilarities across bacterial species under the primary CF-identification thresholds. Twenty independent stochastic subsamplings were performed, with each subsampling retaining 0.01% of all pairwise species comparisons and using random seeds 2027–2046. Spearman correlation coefficients and corresponding *P* values are shown in each panel. Correlations remained consistently positive across the 20 subsamplings, with a median Spearman’s rho of 0.52 and a range of 0.46–0.58, supporting the reproducibility of the observed phylogeny-associated CF repertoire pattern.

**Figure S2. Differential abundance of CFs and repeated cross-validation performance of CF- and taxonomy-based classifiers across intestinal regions.** (A) Heatmap showing log₂ fold changes in CF abundance for pairwise comparisons among acute-infection, chronic-infection, and control groups within the cecum and small intestine. Differential abundance was assessed using DESeq2 with unnormalized raw integer counts and the poscounts size-factor estimation method. *P* values were adjusted using the Benjamini–Hochberg procedure. Red asterisks indicate statistically significant differences after multiple-testing correction. (B) Boxplots showing the distributions of macro-AUC and accuracy obtained from 100 repeated stratified ten-fold cross-validation runs for All-CF, species-level taxonomic, and genus-level taxonomic random forest models in the cecum and small intestine. Each tissue-specific dataset contained 30 balanced samples, including 10 control, 10 acute-infection, and 10 chronic-infection samples. Points represent individual cross-validation repetitions. Dashed horizontal lines indicate chance-level performance for macro-AUC (0.5) and accuracy (1/3).

**Figure S3.** **Sensitivity of species-level CF repertoire analyses to alternative sequence-coverage thresholds.** Species-level CF presence/absence matrices were reconstructed using relaxed paired query/subject coverage cutoffs while the species-prevalence cutoff was fixed at 50%. (A–E) Paired query/subject coverage cutoffs of 50/50. (F–J) Paired query/subject coverage cutoffs of 60/60. Within each threshold setting, panels show the Jaccard-based PCoA of species-level CF profiles, taxonomic PERMANOVA adjusted *R*² values, the association between phylogenetic distance and CF-based Jaccard dissimilarity, the Calinski–Harabasz index for PAM cluster selection, and the resulting two-cluster PAM configuration, respectively.

**Figure S4.** **Sensitivity of species-level CF repertoire analyses to alternative species-prevalence thresholds.** Species-level CF presence/absence matrices were reconstructed using alternative species-prevalence cutoffs while the paired query/subject coverage cutoffs were fixed at 70/70. (A–E) Species-prevalence cutoff of 30%. (F–J) Species-prevalence cutoff of 70%. Within each threshold setting, panels show the Jaccard-based PCoA of species-level CF profiles, taxonomic PERMANOVA adjusted *R*² values, the association between phylogenetic distance and CF-based Jaccard dissimilarity, the Calinski–Harabasz index for PAM cluster selection, and the resulting two-cluster PAM configuration, respectively.

**Figure S5. Prevalence-matched random KO gene-set null model for the association between phylogenetic distance and gene-content dissimilarity.** A total of 999 random KO gene sets were generated, with each set containing the same number of features as the observed CF set and matched to the species-prevalence distribution of the CF families. For each random KO set, Jaccard dissimilarities were calculated using the same fixed set of 50,000 species pairs as in the CF analysis, and Spearman correlations with patristic distance were determined. The histogram shows the null distribution of Spearman correlation coefficients obtained from the random KO sets. The red vertical line indicates the observed correlation between CF-based Jaccard dissimilarity and phylogenetic distance (Spearman’s rho = 0.524). The median correlation of the null distribution was 0.606, and the observed CF correlation did not exceed the background expectation (one-sided empirical *P* = 0.898).

**Figure S6. Complementary validation of species-level CF repertoire clustering.** (A) Average silhouette width for Partitioning Around Medoids (PAM) clustering solutions with candidate cluster numbers ranging from *k* = 2 to *k* = 10. The average silhouette width was slightly higher at *k* = 3 than at *k* = 2, indicating that both solutions captured related and partially overlapping CF repertoire structures. (B) Agreement between the PAM and divisive hierarchical clustering analysis (DIANA) assignments for the two-cluster solution. Cell values indicate the number and percentage of species assigned to each combination of PAM and DIANA clusters. Agreement between the two clustering methods was quantified using the adjusted Rand index (ARI = 0.447).

**Figure S7. Multivariate dispersion of CLR-transformed CF profiles across infection groups in the cecum and small intestine.** Boxplots with individual sample points show bias-adjusted Aitchison distances to the group spatial median for control, acute-infection, and chronic-infection samples in the cecum (A) and small intestine (B). Homogeneity of multivariate dispersion was evaluated using PERMDISP with 999 permutations. Multivariate dispersion differed significantly among the cecal groups (*F* = 12.434, *P* = 0.001). Pairwise comparisons showed significant differences between the control and acute groups (BH-adjusted *P* = 0.022), the control and chronic groups (BH-adjusted *P* = 0.022), and the acute and chronic groups (BH-adjusted *P* = 0.006). The acute group showed the greatest mean dispersion, the chronic group showed the lowest dispersion, and the control group was intermediate. No significant difference in multivariate dispersion was detected among the small-intestinal groups (*F* = 0.128, *P* = 0.899).
